# Patient-Reported Outcomes of Maxillomandibular Surgery for Obstructive Sleep Apnea Treatment: A Scoping Review

**DOI:** 10.3390/jcm13051232

**Published:** 2024-02-21

**Authors:** Inês Francisco, Catarina Nunes, Anabela Baptista Paula, Filipa Marques, Madalena Prata Ribeiro, Mariana McEvoy, Mariana Santos, Catarina Oliveira, Carlos Miguel Marto, Gianrico Spagnuolo, Eunice Carrilho, Raquel Travassos, Francisco Vale

**Affiliations:** 1 Institute of Orthodontics, Faculty of Medicine, University of Coimbra, 3000-075 Coimbra, Portugal; mcal9497@gmail.com (C.N.); anabelabppaula@sapo.pt (A.B.P.); filipa.p.s.marques@gmail.com (F.M.); madalenaprata@hotmail.com (M.P.R.); marianamcevoy@gmail.com (M.M.); mariana0santos00@gmail.com (M.S.); catarinafoliveira6@gmail.com (C.O.); raqueltravassos.91@gmail.com (R.T.); fvale@fmed.uc.pt (F.V.); 2 Coimbra Institute for Clinical and Biomedical Research (iCBR), Area of Environment Genetics and Oncobiology (CIMAGO), Faculty of Medicine, University of Coimbra, 3000-075 Coimbra, Portugal; cmiguel.marto@uc.pt (C.M.M.); eunicecarrilho@gmail.com (E.C.); 3 Laboratory for Evidence-Based Sciences and Precision Dentistry, University of Coimbra, 3000-075 Coimbra, Portugal; 4 Centre for Innovative Biomedicine and Biotechnology (CIBB), University of Coimbra, 3000-075 Coimbra, Portugal; 5 Clinical Academic Center of Coimbra (CACC), 3030-370 Coimbra, Portugal; 6 Institute of Integrated Clinical Practice, Faculty of Medicine, University of Coimbra, 3004-531 Coimbra, Portugal; 7 Institute of Experimental Pathology, Faculty of Medicine, University of Coimbra, 3004-531 Coimbra, Portugal; 8 Department of Neurosciences, Reprodutive and Odontostomatological Sciences, University of Naples “Federico II”, 80138 Naples, Italy

**Keywords:** dental patient-reported outcome, oral health-related quality of life, OSA, orthognathic surgery, orthodontics

## Abstract

**(1) Background:** The present scoping review aims to scrutinize all existing patient-reported outcomes and assess the perspectives of obstructive sleep apnea patients after maxillomandibular surgery. **(2) Methods:** The review was carried out according to the extensions for scoping reviews using the PRISMA-ScR guidelines. Several databases were used to carry out the initial search. This study included randomized controlled trials, cohort studies, cross-sectional and case-control studies. The included studies considered patients with obstructive sleep apnea who were submitted to orthognathic surgery as the main subjects, and the patient’s perception of quality of life, satisfaction, treatment experience and side effects were assessed. **(3) Results:** From 1407 examined articles, a total of 16 were included. Most of the included studies used more than one questionnaire to assess quality of life, except for five articles. The most commonly referred instruments were the Epworth Sleepiness Scale, SF-36, the Functional Outcomes of Sleep and Ottawa Sleep Apnea. The most commonly assessed outcomes were sleep quality, daytime function, facial aesthetics, dental function and emotional health. **(4) Conclusions:** The number of variables that can be evaluated from a patient’s perspective are endless, as are the tools available to assess them. Not all of these tools, which are generally questionnaires, assess all the various outcomes, and some do not compare the pre- and post-surgical situations. Most of them are generic and lack specificity for obstructive sleep apnea.

## 1. Introduction

The American Academy of Sleep Medicine updated the international classification of sleep disorders in June 2023 to define obstructive sleep apnea (OSA) as the presence of one or more of the following symptoms: fatigue, insomnia, sleepiness or other symptoms that alter sleep-related quality of life [1]. OSA is classified as a sleep-related breathing disorder, and it affects between 2% and 5% of the pediatric population and 9% to 38% of the adult population worldwide [2,3,4]. This pathology results in a partial or total obstruction of the upper airway while the individual sleeps, with repeated respiratory arrests during this period. Its etiology is the focus of debate as it is thought to be multifactorial and may vary according to sex and age [2]. The associated pathophysiological factors can vary from anatomical (skeletal or soft tissue) to biomechanical, such as increased airflow resistance [5]. Other predisposing factors such as obesity, some genetic syndromes and respiratory and inflammatory pathologies should also be considered when discussing OSA. This condition can hinder development in patients of pediatric age, resulting in a reduced intellectual performance in adulthood [2,6,7]. Scientific evidence suggests that OSA is a risk factor for other pathologies like high blood pressure, cardiac arrhythmia, ischemic heart disease, metabolic disorders and cognitive dysfunction, among others [8,9,10].

When the first signs and symptoms appear, the diagnosis and treatment of OSA should be led by a multidisciplinary team with the aid of various examinations and validated questionnaires [11]. There are numerous questionnaires available for OSA screening in both adults and children. The most common for adults are the Epworth Sleepiness Scale (ESS) [12], the STOP-Bang questionnaire [13] and the Berlin questionnaire [14]. For children, the Pediatric Sleep Questionnaire (PSQ) is the most used questionnaire [15]. Due to its simplicity, the ESS is the most frequently used survey; however, it also has a lower diagnostic value because it measures the general level of sleepiness. The STOP-Bang and Berlin questionnaires, on the other hand, determine the risk of sleep apnea. Both questionnaires assess the signs, symptoms and risk factors of OSA, including snoring, drowsiness, witnessed apnea, obesity and hypertension, among others. The PSQ is a targeted questionnaire used mostly to assess behavior and cognitive performance in pediatric populations. For a thorough diagnosis, other tests, such as an overnight polysomnography (PSG) or an overnight home sleep apnea test (HSAT), should also be performed [11,16].

After a positive diagnosis with OSA, the patient is then categorized using the Apnea-Hypopnea Index (AHI), which categorizes patients according to the number of breathing events that occur per hour during sleep, with the following classification levels: mild (AHI = 5–15 events per hour; oxyhemoglobin saturation (SpO_2_)—86–91%); moderate (AHI = 15–30 events per hour; SpO_2_ = 76–85%); severe (AHI > 30 events per hour; SpO2 ≤ 75%) [17]. Similar to its diagnosis, OSA treatment should also be multidisciplinary, with the gold standard in adulthood being Positive Airway Pressure therapy using Continuous Positive Airway Pressure (CPAP) devices [11,16]. This therapy is effective in eliminating respiratory obstruction events and improves oxygen saturation during sleep. Nonetheless, tolerance and compliance to the therapy are generally reduced [18,19]. In the pediatric population diagnosed with OSA, rapid maxillary expansion (RME), mandibular advancement devices (MADs) and reverse traction extraoral appliances (RPHG) are the preferred treatment options for mild-to-moderate cases as they seem to lessen some of the symptoms temporarily. There are several literature reviews that report a decrease in the Apnea-Hypopnea Index (AHI) of about six points after the use of RMEs and MADs [20,21,22]. In individuals with severe OSA who are intolerant to CPAP, orthodontic–surgical treatment may be an option to take into consideration. The maxillomandibular advancement surgery increases the size of the nasopharyngeal, retropalatal and hypopharyngeal airways due to the physical expansion of the facial skeletal structure. The advancement of the maxilla and mandible increases tension in the pharyngeal soft tissues, the base of the tongue and the soft palate, thereby enlarging the mediolateral and anteroposterior dimensions of the upper airway. Regarding surgical treatment for OSA, a recent meta-analysis demonstrated that this treatment presented a mean decrease in the AHI from 63.9 to 9.5 events/hour, with a combined surgical success rate of 86.0% and an overall OSA treatment success rate of 43.2% [23]. There are other studies that demonstrate the high effectiveness of this surgical approach, showing that it reduces the frequency of respiratory events and drowsiness, increasing overall quality of life [10,24]. Furthermore, when there is a narrowing of the oropharynx, velopharynx and/or hypopharynx and maxillary retrognathia or skeletal hypoplasia, surgery is the most adequate therapeutic option [10,25]. The pre-operative severity of OSA is the most reliable predictor of the outcome effect, dimension and likelihood of surgical success. The more severe the OSA, the more benefits are reaped [26]. Less severe patients achieve an improvement in the AHI or RDI (respiratory disturbance index) of lower magnitude post-operatively but have a higher chance of successful treatment. Patients with less severe cases of RDI and AHI (despite previous treatments such as uvulopalatopharyngoplasty, partial glossectomy and/or nasal surgery) are highly likely to benefit from surgical maxillomandibular advancement [26].

Despite evidence supporting the success of orthognathic surgery as a treatment for OSA, it is also important to consider patient-reported outcomes (PROs). Evaluating the adaptability, adverse effects, impact of the surgery on the quality of sleep and quality of life of patients and their families is of the utmost importance. This review aims to evaluate the current literature regarding Patient-Reported Outcomes Measurements (PROMs) for children and adults with OSA who have undergone maxillomandibular surgery, specifically quality of life (QoL), adverse effects, patient satisfaction, overall experience with the treatment and the perception of occlusal or dental changes after treatment. The present scoping review intends to scrutinize all existing PROMs to assess the perspective of the OSA patient after maxillomandibular surgery.

## 2. Materials and Methods

The present review was carried out according to the extensions for scoping reviews using the Preferred Reporting Items for Systematic Reviews and Meta-Analysis (PRISMA-ScR) criteria.

### 2.1. Research Question

The research question was chosen considering the patient’s perspective regarding expectations, satisfaction and quality of life after orthognathic surgery. The research question is described in Table 1.

The aim of this study was to evaluate the perspectives of OSAS patients after undergoing orthognathic surgery.

### 2.2. Database Search Protocol

For the present scoping review, a search was carried out using several databases, such as Medline (PubMed), all Web of Science databases, Embase and Cochrane. Table 2 describes the search keys, which were used on 7 March 2023.

Beyond the described databases, a search was also conducted in the gray literature on the following websites: OpenGrey Europe (https://opengrey.eu, accessed on 10 March 2023) and ProQuest (https://www.proquest.com, accessed on 10 March 2023).

### 2.3. Analysis of Eligibility Criteria, Selection of Studies and Data Collection

This study exclusively included randomized controlled trials (RCT), cohort studies (prospective and retrospective), cross-sectional studies and case–control studies. Inclusion and exclusion criteria were established based on the research question. The inclusion criteria adopted during the development of this systematic review were as follows: (1) patients with OSAS submitted orthognathic surgery and (2) studies that evaluated patients’ perceptions regarding quality of life (QoL), namely satisfaction and treatment experience, as well as side effects.

Other types of papers, such as umbrella reviews, systematic reviews, case series studies, case–control editorials, conference abstracts, book chapters, guidelines, protocols, and opinion papers were excluded from the analysis. Studies including patients with systematic diseases, known genetic syndromes, and participants without an OSAS diagnosis were also excluded.

Two calibrated researchers (M.M. and C.O.) were responsible for article selection, following predefined inclusion and exclusion criteria. In instances where there was disagreement, a calibrated investigator (C.N.) assessed the articles in question. The duplicated citations were removed using automated and manual tools. The initial evaluation of studies involved screening their titles and abstracts. Those that met the inclusion criteria proceeded to undergo a thorough reading in full.

The included studies were scrutinized, and the following data were gathered: author; year of publication; sample size; sex; mean age of patients; type of orthognathic surgery (unimaxilar or bimaxilar); evaluated parameters; evaluation instruments and their description (PROM’s); and final observations.

## 3. Results

### 3.1. Study Selection

**Figure 1:**
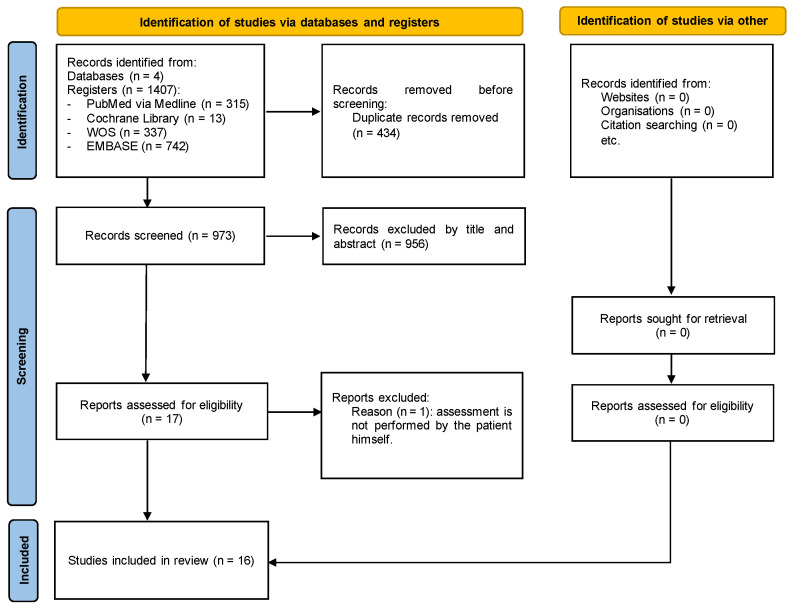
Flow diagram.

The search, screening, and eligibility processes are described in Figure 1. Initially, 1407 articles were retrieved from the search, none of which were cross-referenced. After removing duplicates, 973 articles were analyzed by title and abstract, resulting in 17 articles being selected for full reading. After fully reading the articles, it was found that one article did not evaluate the patient’s perspective, as the quality of life assessment was carried out by family members, leading to the exclusion of this article. Thus, sixteen articles were included in this review (Figure 1).

### 3.2. Characteristics of the Included Studies

Sixteen articles assessed the impacts of orthognathic surgery on satisfaction and patient quality of life. The year of publication ranged from 2004 [27] to 2023 [28], given that five were published in 2020 [20,29,30,31] and three in 2022 [32,33,34]. Regarding the type of study, eight were retrospective cohort studies, four were cohort studies, three were prospective cohort studies and one was a retrospective case–control study. The sample size ranged from ten [34] to fifty-seven [27], resulting in a total sample of *n* = 413. The average age of patients was between 34.75 ± 11.33 [32] and 59.1 ± 11.7 years [29]. Apart from one study that did not mention the patients’ sex [35], all studies included more men than women. Regarding the instruments, five articles used a single questionnaire [27,29,30,36,37], while eleven employed multiple questionnaires, with the most extensive one using six [38]. The most commonly referred instruments were the Epworth Sleepiness Scale (nine studies) [27,28,31,34,35,37,38,39,40,41], SF-36 (four studies) [32,38,39,42], Functional Outcomes of Sleep (three studies) [31,39,41], Ottawa Sleep Apnea (two studies) [36,39], and Rustemever’s method (two studies) [32,33]. The most evaluated outcomes were sleep quality, daytime function, facial aesthetics, dental function and emotional health. Table 3 reports a summary of the characteristics of the included studies.

## 4. Discussion

The aim of this scoping review was to report on the current state of the art regarding the tools available to assess quality of life from the patient’s perspective after orthognathic surgery to manage OSAS. The qualitative analysis of the studies available in the literature allowed for a better understanding and evaluation of the studies published on this topic.

From the patient’s perspective, several outcomes can be assessed to rate quality of life. These can be related to aesthetics, function, mental health, sleep quality and post-operative condition. Regarding aesthetic outcomes, these are directly related to facial aesthetics and concern the facial changes resulting from orthognathic surgery. Mental health outcomes focus on general well-being, emotional status, mood, anxiety and depression. In relation to function, patients assess their general daily productivity, level of activity and focus, mobility, chewing, phonation, swallowing and intimacy, more specifically the quality of sexual life. Sleep quality includes outcomes such as insomnia, daytime sleepiness, frequent nocturnal diuresis and snoring. And, finally, some tools assess the post-operative condition, allowing the patient to describe their post-surgical recovery regarding the presence of signs and/or symptoms resulting from the surgery, such as pain or discomfort.

The best way to improve outcome reports is to develop and apply a core outcome set (COSs), that is, the minimum set of measurable and relevant outcomes that should be measured and reported in all disease-specific clinical trials [43]. Currently, the Core Outcome Measures in Effectiveness Trials (COMET) initiative promotes the development and implementation of COSs for the selection of health measurement instruments (COSMIN) [44]. However, on the topic of obstructive sleep apnea, there is still no defined COS, which makes the standardization of studies difficult. The heterogeneity in outcomes across studies poses significant challenges for drawing clear conclusions or generalizing findings, making informed decisions more complicated. This variability complicates the development of meta-analyses and consistent policies or guidelines, leading to inefficient resource allocation. To address these issues, standardization efforts are crucial. This includes establishing standardized outcome measures, protocol standardization, promoting data sharing and collaboration, utilizing advanced meta-analysis techniques and implementing quality improvement initiatives. These strategies will enable researchers to enhance research reliability and reproducibility, thus resulting in knowledge advancement and improved decision-making. This scoping review produced a synthesis of an existing body of literature, which can help to close the existing gap, since it verified the most evaluated results: sleep quality, daytime function, facial aesthetics, dental function and emotional health. In addition, the main focus of this review was assessing the outcomes from the patients’ perspectives, subsequently determining the relevance of the evaluated outcomes.

In the sixteen included studies, fifteen different tools for assessing quality of life from the patient’s perspective can be found, most of which consist of questionnaires completed by patients themselves before and/or after orthognathic surgery. Out of all the tools, five stand out: the Epworth Sleepiness Scale (ESS), SF-36, the Functional Outcomes of Sleep Questionnaire (FOSQ), Rustemeyer’s Questionnaire and the Ottawa Sleep Apnea Questionnaire (OSA-Q).

The ESS was used the most in the majority of included studies, having been employed in about 55% of the studies. It consists of a standardized and validated questionnaire that measures the level of daytime sleepiness. It consists of eight questions about subjective sleepiness in eight different everyday situations, each rated on a scale of 0 to 3, with a maximum score of 24. Normal values range between 2 and 10, while scores above 10 indicate a high level of excessive or pathological daytime sleepiness. In almost all studies, the questionnaire was administered at two different points in time: pre-surgery and post-surgery, allowing for the determination of long-term changes in subjective sleepiness after surgery. According to Matthew T Scharf [45], although the ESS is widely adopted and considered to be a useful tool for the assessment of excessive sleepiness, it should be applied and interpreted with caution within the appropriate clinical context, and it should be complemented with other assessment tools, especially when the test is negative [45,46]. The main advantages of the ESS are low cost, easy access and excellent validation. However, Matthew T Scharf [45] found some disadvantages, such as variability in the results obtained when the test was repeated and the fact that it measures different variables in different populations, meaning that a specific score in one group may present disparate variables when compared to a similar score in another group. Recently, Gonçalves et al. reinforced the need for further studies to investigate which variables may be the cause of the observed variability, making it important to include not only psychometric studies but also empirical studies [47].

The SF-36 questionnaire was the second most used tool in the included studies (25% of studies). Similar to the ESS, it is a questionnaire that needs to be completed at two different times: pre- and post-surgery, allowing for the measurement and comparison of surgical impacts on quality of life in patients with OSAS. This questionnaire assesses two components: physical and mental. The physical component includes the physical function, physical role, bodily pain and general health perception scales, while the mental component includes the vitality, social functioning, emotional role and general mental health scales. Scores range from 0 to 100, with 100 representing the highest level of function. These two components are divided into eight domains. The final scores in each domain represent an average of the scores obtained in the questions addressing that specific domain [48]. The frequent use of this tool may be related to some advantages such as its generic nature for assessing health status, its easy administration and comprehension and the fact that it can be used in groups of any age, pathology, treatment, ethnicity or gender. In fact, this tool is considered in the literature to be a reliable, valid and responsive method for a variety of medical diagnoses. Regarding OSAS, this method has been reported as sensitive to determine the effects of this pathology.

The third most commonly used tool was the Functional Outcomes of Sleep Questionnaire (FOSQ), appearing in 19% of the included studies. Similar to the questionnaires mentioned above, this survey is also conducted pre- and post-surgery. The FOSQ consists of 30 questions divided into 5 domains: activity level, vigilance, intimacy and sexual relationships, general productivity and social outcome. The scores for each domain are added together to give a total score, with a maximum of 20 points. A low score represents dysfunction due to excessive sleepiness during the day [31,48]. A major difference between this tool and the others is the fact that it assesses the sexual component. However, although this is an advantage, it can also sometimes be considered prejudicial, as some people may find the questions of this domain offensive and/or embarrassing, which may induce bias in the results [31,49].

In the present study, the three questionnaires mentioned above stood out. However, two others also stood out: the Ottawa Sleep Apnea Questionnaire (OSA-Q) and Rustemeyer’s Questionnaire. Rustemeyer’s Questionnaire, unlike the previously described tools, is performed only at one point, which is during the post-surgical period. Thus, it is only considered to be a post-operative tool. It consists of six questions, and the score ranges from 0 to 10. It aims to assess the patient’s overall satisfaction; changes in quality of life, aesthetics and masticatory function after undergoing orthognathic surgery; and the opinions of family and friends [32]. Finally, the OSA-Q consists of a questionnaire comprised of 38 questions regarding quality of life on a five-point Likert scale. The first 30 questions are related to sleep quality, daytime function and physical, mental, emotional and sexual health. However, as with the FOSQ, there is also a low response rate for questions related to intimacy and sexual health; so, in the study by Butterfield et al., these questions were made optional in order to encourage patients to complete the questionnaire [36] The remaining eight questions on the Likert scale are mainly aimed at assessing the impacts of side effects of orthognathic surgery on quality of life, taking into account surgical recovery and masticatory function [36].

Therefore, it can be noted that the number of outcomes that can be assessed from the patient’s perspective is countless, as are the tools available to assess them. Nevertheless, not all these tools, which are mostly questionnaires, assess all the various domains of outcomes, and some do not compare the pre-surgical situation with the post-surgical one. In future studies, it would be important to create a questionnaire that compiles all questions related to all outcomes and standardizes the assessment of quality of life in patients diagnosed with OSAS undergoing orthognathic surgery. Despite the variation in outcomes, most studies included were classified as good quality (scoring ≥ 7 points), facilitating a clearer interpretation of the results. This study could be the starting point for a more patient-centered precision medicine. In fact, patient-reported outcomes play a crucial role in clinical practice, patient care and future research directions in healthcare. Understanding the patient’s perspective on their health status, symptoms and quality of life provides valuable information about the effectiveness of treatments and interventions. Clinicians use these outcomes to tailor patient care plans, monitor treatment progress and make informed decisions about the most appropriate interventions for individual patients. Additionally, PROs enable OSA patients to actively participate in their care by articulating their concerns and preferences, fostering shared decision-making between patients and healthcare providers. Moreover, PRO data contribute to the advancement of research by identifying areas for improvement in OSA treatment, guiding the development of new interventions and evaluating the impact of interventions on patient outcomes. As healthcare continues to evolve to take on more patient-centered characteristics, the integration of PROs into OSA clinical practice and research will become increasingly essential for optimizing patient outcomes and enhancing the overall quality of care.

The best method for improving outcome reports is to develop and apply a core outcome set (COS), that is, the minimum set of measurable and relevant outcomes that should be measured and reported in all disease-specific clinical trials. However, for OSA, there is still no defined COS, which made it difficult to standardize the studies. Researchers should join the COMET Initiative, as it is a platform that allows the gathering of relevant resources, both applied and methodological, in order to facilitate the exchange in ideas and information and encourage methodological research in this area. Notwithstanding its limitations, this scoping review helps to fill the existing literature gap, as it has identified the five most-evaluated outcomes, which were sleep quality, daytime function, facial aesthetics, dental function and emotional health. It would be important to create a specific questionnaire for OSA/Orthognathic Surgery that compiles all questions related to all outcomes and standardizes the assessment of quality of life. Future studies should define the key results for characterizing quality of life, allowing for the development of a complete and effective questionnaire.

## 5. Conclusions

The number of outcomes that can be assessed from the patient’s perspective is countless, as are the tools available to assess them. The most evaluated outcomes were sleep quality, daytime function, facial aesthetics, dental function and emotional health. Collaborative endeavors from researchers are imperative to foster the widespread and uniform adoption of patient outcome reports in OSA research.

## Figures and Tables

**Table 1:** PICO question.

Research Question	
Population	Patients diagnosed with OSAS
Intervention	Patients who underwent orthognathic surgery
Outcome	Patients’ perspectives (PROMs)

**Table 2:** Search keys of several Databases.

Data Bases	Search Keys		
PubMed	(“Sleep Apnea Syndromes”[Mesh] OR “Sleep Apnea*” OR “Apnea Syndrome, Sleep” OR “Apnea Syndromes, Sleep” OR “Sleep Hypopnea*” OR “Hypopnea, Sleep” OR “Hypopneas, Sleep” OR “Apnea, Sleep” OR “Apneas, Sleep” OR “Hypersomnia with Periodic Respiration” OR “Sleep-Disordered Breathing” OR “Breathing, Sleep-Disorder*” OR “Sleep Disordered Breathing” OR “Sleep-Disorder Breathing” OR “Sleep Disorder Breathing” OR “OSA” OR “OSAS”) AND (“Orthognathic Surgery”[Mesh] OR “Orthognathic Surger*” OR “Surgery, Orthognathic” OR “Surgeries, Orthognathic” OR “Orthognathic Surgical Procedures”[Mesh] OR “Orthognathic Surgical Procedure*” OR “Procedure, Orthognathic Surgical” OR “Procedures, Orthognathic Surgical” OR “Surgical Procedure, Orthognathic” OR “Surgical Procedures, Orthognathic” OR “Jaw Surger*” OR “Surgeries, Jaw” OR “Surgery, Jaw” OR “Maxillo-Mandibular Surger*” OR “Maxillo Mandibular Surger*” OR “Surgeries, Maxillo-Mandibular” OR “Surgery, Maxillo-Mandibular” OR “Surgeries, Maxillofacial Orthognathic” OR “Surgery, Maxillofacial Orthognathic”). Filters: languages—EN, SP, FR, and PT.		
All Web of Science Databases	(“Sleep Apnea*” OR “Apnea Syndrome, Sleep” OR “Apnea Syndromes, Sleep” OR “Sleep Hypopnea*” OR “Hypopnea, Sleep” OR “Hypopneas, Sleep” OR “Apnea, Sleep” OR “Apneas, Sleep” OR “Hypersomnia with Periodic Respiration” OR “Sleep-Disordered Breathing” OR “Breathing, Sleep-Disordered” OR “Sleep Disordered Breathing” OR “OSA” OR “OSAS”) AND (“Orthognathic Surger*” OR “Surgery, Orthognathic” OR “Surgeries, Orthognathic” OR “Orthognathic Surgical Procedure*” OR “Procedure, Orthognathic Surgical” OR “Procedures, Orthognathic Surgical” OR “Surgical Procedure, Orthognathic” OR “Surgical Procedures, Orthognathic” OR “Jaw Surger*” OR “Surgeries, Jaw” OR “Surgery, Jaw” OR “Maxillo-Mandibular Surger*” OR “Maxillo Mandibular Surger*” OR “Surgeries, Maxillo-Mandibular” OR “Surgery, Maxillo-Mandibular” OR “Surgeries, Maxillofacial Orthognathic” OR “Surgery, Maxillofacial Orthognathic”) (Topic) and English or French or Spanish or Portuguese (Languages) and Review Article or Abstract or Meeting or Letter or Editorial Material or Patent or Book (Exclude—Document Types). Filters: languages—EN, SP, FR, and PT.		
Embase	(“sleep apnea*”: ti,ab,kw OR “apnea syndrome, sleep”: ti,ab,kw OR “apnea syndromes, sleep’”: ti,ab,kw OR “sleep hypopnea*”:ti,ab,kw OR “hypopnea, sleep”: ti,ab,kw OR “hypopneas, sleep”: ti,ab,kw OR “apnea, sleep”: ti,ab,kw OR “apneas, sleep”: ti,ab,kw OR “hypersomnia with periodic respiration”: ti,ab,kw OR “sleep disordered breathing”/exp OR “sleep-disordered breathing”: ti,ab,kw OR “breathing, sleep disordered”: ti,ab,kw OR osa: ti,ab,kw OR osas: ti,ab,kw) AND (“orthognathic surgery”/exp OR “orthognathic surger*”: ti,ab,kw OR “surgery, orthognathic”: ti,ab,kw OR “surgeries, orthognathic”: ti,ab,kw OR “orthognathic surgical procedure*”: ti,ab,kw OR “procedure, orthognathic surgical”: ti,ab,kw OR “procedures, orthognathic surgical”: ti,ab,kw OR “surgical procedure, orthognathic”: ti,ab,kw OR “surgical procedures, orthognathic”: ti,ab,kw OR “jaw surger*”: ti,ab,kw OR “surgeries, jaw”: ti,ab,kw OR “surgery, jaw”: ti,ab,kw OR “maxillomandibular surger*”: ti,ab,kw OR “maxillo mandibular surger*”: ti,ab,kw OR “surgeries, maxillo-mandibular”: ti,ab,kw OR “surgery, maxillo-mandibular”: ti,ab,kw OR “maxillofacial orthognathic surger*”: ti,ab,kw OR “orthognathic surgeries, maxillofacial”: ti,ab,kw OR “orthognathic surgery, maxillofacial”: ti,ab,kw OR “surgeries, maxillofacial orthognathic”: ti,ab,kw OR “surgery, maxillofacial orthognathic”: ti,ab,kw) AND ([english]/lim OR [french]/lim OR [portuguese]/lim OR [spanish]/lim) AND ([article]/lim OR [article in press]/lim OR [data papers]/lim OR [letter]/lim) Filters: languages—EN, SP, FR, and PT.		
Cochrane	ID	Search	Hits
	#1	MeSH descriptor: [Sleep Apnea Syndromes] explode all trees	3389
	#2	“sleep apnea”	8129
	#3	“sleep apneas”	111
	#4	“apnea syndrome, sleep”	14
	#5	“apnea syndromes, sleep”	869
	#6	“sleep hypopnea”	0
	#7	“sleep hypopneas”	1
	#8	“hypopnea, sleep”	7
	#9	“hypopneas, sleep”	0
	#10	“apnea, sleep”	412
	#11	“apneas, sleep”	3
	#12	hypersomnia with periodic respiration	0
	#13	“sleep-disordered breathing”	3381
	#14	“breathing, sleep-disordered”	4
	#15	“sleep disordered breathing”	3381
	#16	OSA	3950
	#17	OSAS	709
	#18	MeSH descriptor: [Orthognathic Surgery] explode all trees	64
	#19	“orthognathic surgery”	601
	#20	“orthognathic surgeries”	29
	#21	“surgery, orthognathic”	40
	#22	“surgeries, orthognathic”	0
	#23	MeSH descriptor: [Orthognathic Surgical Procedures] explode all trees	279
	#24	“orthognathic surgical procedure”	5
	#25	“orthognathic surgical procedures”	199
	#26	“procedure, orthognathic surgical”	0
	#27	“procedures, orthognathic surgical”	8
	#28	“surgical procedure, orthognathic”	0
	#29	“surgical procedures, orthognathic”	7
	#30	“jaw surgery”	65
	#31	“jaw surgeries”	4
	#32	“surgeries, jaw”	2
	#33	“surgery, jaw”	11
	#34	“maxillo-mandibular surgery”	0
	#35	“maxillo-mandibular surgeries”	0
	#36	“maxillo mandibular surgery”	0
	#37	“maxillo mandibular surgeries”	0
	#38	“surgeries, maxillo-mandibular”	0
	#39	“surgery, maxillo-mandibular”	0
	#40	“maxillofacial orthognathic surgery”	1
	#41	“maxillofacial orthognathic surgeries”	0
	#42	“orthognathic surgeries, maxillofacial”	0
	#43	“orthognathic surgery, maxillofacial”	3
	#44	“surgeries, maxillofacial orthognathic”	0
	#45	“surgery, maxillofacial orthognathic”	0
	#46	(#1 OR #2 OR #3 OR #4 OR #5 OR #6 OR #7 OR #8 OR #9 OR #10 OR #11 OR #12 OR #13 OR #14 OR #15 OR #16 OR #17) AND (#18 OR #19 OR #20 OR #21 OR #22 OR #23 OR #24 OR #25 OR #26 OR #27 OR #28 OR #29 OR #30 OR #31 OR #32 OR #33 OR #34 OR #35 OR #36 OR #37 #38 OR #39 OR #40 OR #41 OR #42 OR #43 OR #44 OR #45)	

**Table 3:** Characteristics of the Included Studies.

Author/ Year	Study Design	Sample Size	Mean Age of Patients	Sex	Instruments	Outcomes
Cillo et al., 2020 [30]	Retrospective cohort	27	59.1 ± 11.7 years old	M = 15 F = 12	Ottawa Sleep Apnea	Sleep quality
						Daytime function
						Physical health
						Emotional health
						Sexual desires
						Functional desires
						Dental function
						Personal Satisfaction
Cillo et al., 2019 [29]	Retrospective cohort	27	59.8 years old	M = 15 F = 12	Modified survey from Cunningham et al. using seven-point visual analog scale	Chewing
						Swallowing food
						Swallowing fluids
						Smiling
						Spitting
						Kissing
						Eating
						Drooling
						Speaking
						Perioral neurosensory
Dattilo, Drooger, et al., 2004 [27]	Prospective cohort	57	47.2 years old	M = 43 F = 14	Epworth Sleepiness Scale	Probability of falling asleep in a variety of situations (sitting, reading, watching TV and driving)
Rossi et al., 2022 [32]	Retrospective cohort	18	44.39 ± 9.43 years old	M = 17 F = 1	- Rustemeyer’s questionnaire - Post-operative quality-of-life questions specific for OSA	Rustemeyer’s questionnaire: -Facial aesthetics -Chewing function -Well-being Post-operative quality-of-life questions specific for OSA: -Quality of sleep -Day time function/activity -Emotional situation -Physical OSAS symptoms -Work activity
Rossi et al., 2022 [33]	Retrospective case-control	61 (21-OSA)	34.75 ± 11.33	M = 33 F = 29	- Rustemeyer’s questionnaire (only after surgery) - SF-36 questionnaire (pre- and post-operatively)	Rustemeyer’s questionnaire: -Facial aesthetics -Chewing function -Well-being SF-36 questionnaire: -Emotional well-being -General health -Health transition -Physical functioning -Role limitations due to physical health -Role limitations due to Emotional problems -Energy/fatigue -Social functioning -Bodily Pain
Boyd et al., 2019 [39]	Prospective cohort	30	45.9 ± 9.8 years	M = 19 F = 11	- Epworth Sleepiness Scale - Functional Outcomes of Sleep Questionnaire - SF-36 questionnaire	Epworth Sleepiness Scale: -Sleepiness Functional Outcomes of Sleep Questionnaire: -General productivity -Social outcome -Activity level -Vigilance -Intimate relationship and sexual activity SF-36 questionnaire: -Physical function -Role physical -Role emotional -Vitality -Mental health -Social function -Bodily pain -General health -Health change
Butterfield et al., 2016 [36]	Retrospective cohort	22	45.9 ± 11.6 years	M = 19 F = 3	- Ottawa Sleep Apnea	Sleep quality
						Daytime function
						Physical health
						Mental and emotional health
						Sexual health
						Recovery
						Dental function
Martin et al., 2022 [34]	Cohort	10	49.9 years	M = 7 F = 3	- Patient-reported outcome measures questionnaire (five-point Likert scale) - Epworth Sleepiness score - 10-point visual analogue scale	Patient-Reported Outcome Measurements (PROMs) questionnaire (five-point Likert scale): -Sleep quality -Daytime sleepiness -Energy levels -Appearance -Daily activities -Mood Epworth Sleepiness score: -Daytime sleepiness 10-point visual analogue scale: -Quality of life
Pottel et al., 2019 [40]	Retrospective cohort	12	43.5 years	M = 10 F = 2	- OSAS questionnaire - The Epworth Sleepiness Scale	OSAS questionnaire: -Headache -Daytime sleepiness -Night-time awakening -Concentration -Frequent nocturnal diuresis -Snoring -Sexual activity -Facial aesthetic -Self-confidence Epworth Sleepiness Scale: -Daytime sleepiness
Goodday, RH et al., 2016 [35]	Retrospective cohort	13	38.6 ± 8.4	NR	- Epworth Sleepiness Scale - General satisfaction survey	Epworth Sleepiness Scale: -Daytime sleepiness General satisfaction survey: -Recommendation -Benefit
Lin, CH et al., 2020 [20]	Cohort	53	35.66 ± 11.66	M = 40 F = 13	- Epworth Sleepiness Scale - Pittsburgh Sleep Quality Index - Insomnia Severity Index - Beck Anxiety Inventory - Beck Depression Index - Short form of quality of life (SF-36)	Epworth Sleepiness Scale: -Daytime sleepiness Pittsburgh Sleep Quality Index: -Quality and patterns of sleep Insomnia Severity Index: -Insomnia Beck Anxiety Inventory: -Anxiety Beck Depression Index: -Depression Short form of quality of life (SF-36): -Physical functioning -Role physical -Bodily pain -General Health -Vitality -Social Functioning -Role emotional -Mental Health
Ruiter MHT et al., 2020 [31]	Cohort	41	55 ± 10	M = 35 F= 20	- EQ-5D-3L - Epworth Sleepiness Scale - Functional Outcomes of Sleep - Visual Analog Scale	EQ-5D-3L: -Mobility -Self-care -Daily activities -Pain/discomfort -Mood Epworth Sleepiness Scale: -Daytime sleepiness Functional Outcomes of Sleep: -Activity level -Vigilance -Intimacy and sexual relationships -General productivity -Social outcomes Visual Analog Scale: Facial appearance
González, MB et al., 2020 [42]	Retrospective cohort	25	46.68	M = 23 F = 2	SF-36 questionnaire	Facial appearance through two questions: - “Do you consider your esthetic change to be positive after surgery?” - “Do you consider your facial profile to be more youthful after surgery?”
Beranger T et al., 2017 [37]	Retrospective cohort	23	45.7	M = 15 F = 8	Epworth Sleepiness Scale	- Facial appearance (modifications of the face, modifications smile, more smiley and youthful appearance)
Boyd, SB et al., 2015 [41]	Cohort	30	50.5 ± 9.6	M = 24 F = 6	- Epworth Sleepiness Scale - Functional Outcome of Sleep Questionnaire - Sleep Apnea Quality of Life Index	Epworth Sleepiness Scale: -Sleepiness Sleep Apnea Quality of Life Index: -Daily functioning -Social interactions -Emotional functioning -Symptoms -Treatment-related symptoms
Abdelwahab, M et al., 2023 [28]	Prospective cohort	31	38 ± 11	M = 28 F = 3	- Standardized Cosmesis and Health Nasal Outcomes Survey - Visual analog scale for nasal function and cosmesis (VAS-F and VAS-C) - Epworth sleepiness scale	Function (obstruction) Cosmesis Epworth sleepiness scale: -Daytime sleepiness

## Data Availability

Since this manuscript is a review, the data in this manuscript were not created, as they already exist in other manuscripts.
